# Tissue‐Specific Expansion of Age‐Associated B Cells via IFN‐γ and IL‐21 Within Salivary Glands in Sjögren Disease

**DOI:** 10.1155/jimr/4221251

**Published:** 2026-03-24

**Authors:** Mari Nishida, Kunihiro Otsuka, Ruka Nagao, Shigefumi Matsuzawa, Aya Ushio, Takaaki Tsunematsu, Keiko Aota, Naozumi Ishimaru

**Affiliations:** ^1^ Department of Oral Pathology, Tokushima University Graduate School of Biomedical Sciences, Tokushima, Japan, tokushima-u.ac.jp; ^2^ Department of Oral and Maxillofacial Disease Control, Tokushima University Graduate School of Biomedical Sciences, Tokushima, Japan, tokushima-u.ac.jp; ^3^ Section of Oral and Maxillofacial Surgery, Division of Maxillofacial Diagnostic and Surgical Sciences, Faculty of Dental Science, Kyushu University, Fukuoka, Japan, kyushu-u.ac.jp; ^4^ Department of Oral Pathology, Graduate School of Medical and Dental Sciences, Institute of Science Tokyo, Tokyo, Japan, tmd.ac.jp

**Keywords:** age-associated B cell (ABC), autoimmune disease, Sjögren disease (SjD)

## Abstract

Sjögren disease (SjD) is an autoimmune disorder that predominantly affects the exocrine glands, and advancing age is recognized as an important risk factor for its development. However, the mechanisms linking age and disease progression remain poorly understood. Age‐associated B cells (ABCs), a subset of B cells that increase with age, have been implicated in autoimmune responses, but their role in SjD pathogenesis has not been fully clarified. In this study, we examined labial salivary glands (LSGs) from 44 SjD patients and 11 non‐SjD sicca controls. T‐bet^+^ CD20^+^ ABCs were detected infiltrating the glands in SjD patients, especially in individuals in their 40s–60s, but were rare in non‐SjD sicca controls. To investigate the underlying mechanisms, we used a SjD mouse model at various ages. ABCs (CD11b^+^ CD95^+^ CD19^+^) began locally accumulating in the SGs from the mature‐adult stage, earlier than in age‐matched controls, while remaining low in cervical lymph nodes (cLNs). To explore the drivers of ABC expansion, we examined the factors involved in ABC differentiation, focusing on interleukin‐21 (IL‐21) and interferon‐gamma (IFN‐γ). This combination of IL‐21 and IFN‐γ upregulated T‐bet expression on B cells in SjD model mice. In situ hybridization (ISH) and flow cytometric analysis revealed that CD4^+^ T cells, especially follicular helper T (Tfh)‐like cells were a major source of IL‐21 in the SGs of mature‐adult‐SjD mice. Additionally, ABCs themselves showed elevated expression of IFN‐γ compared to other immune cells, indicating an autocrine mechanism promoting their expansion. Our findings suggest that ABCs accumulate in the SGs of SjD patients and model mice through IL‐21 signaling from CD4^+^ T cells and autocrine IFN‐γ activity. This localized expansion may contribute to autoimmune tissue damage. These results provide new insights into how aging‐associated immune changes may drive the development and progression of SjD, offering potential targets for therapeutic intervention.

## 1. Introduction

Sjögren disease (SjD) is an autoimmune disorder that affects exocrine glands, such as the salivary glands (SGs). CD4^+^ T cells infiltrate the SGs at an early stage, followed by the expansion of B cells, which play a crucial role in exacerbating the disease [1, 2]. However, it remains unclear how B cells expand during the later stages and how they contribute to autoimmune pathology.

Aging is a known risk factor for the onset of autoimmune diseases, including SjD [1–4]; however, the molecular mechanisms linking aging and disease development remain poorly understood. Recent studies have elucidated age‐related changes in various immune cell populations [5–9]. Among these, age‐associated B cells (ABCs) represent a subset of B cells that increase with aging. ABCs are characterized by the expression of T‐box transcription factor (T‐bet), CD11b, CD11c, and CD95 [8, 9]. These cells have the potential to differentiate into antibody‐secreting cells upon antigen exposure [8–10]. Additionally, ABCs can produce interferon‐gamma (IFN‐γ) and promote inflammation [11–13], suggesting their multifaceted roles in the adaptive immune response.

Several studies have indicated that ABCs in lymphoid tissues and peripheral blood contribute to the pathogenesis of autoimmune diseases, either by promoting autoantibody production or by enhancing inflammatory responses [8–10, 14–16]. However, the role of ABCs in the target tissues, such as the SGs, remains poorly defined.

ABCs are thought to arise from naïve B cells upon exposure to interleukin‐21 (IL‐21) and IFN‐γ [17–19]. Nevertheless, the exact mechanism by which ABCs are generated remains uncertain [14]. Given the heterogeneity within the ABC population, it is hypothesized that their differentiation does not occur uniformly. For example, in a lymphocytic choriomeningitis virus (LCMV) infection mouse model, some naïve B cells exposed to IL‐21 in the draining lymph nodes eventually differentiated into ABCs in the liver under the influence of IFN‐γ [20]. These findings suggest that tissue‐specific cytokine signals may play a role in ABC differentiation, though this mechanism has not been fully explored in the context of autoimmune disease.

To investigate how ABCs contribute to the pathogenesis of SjD, we used female thymectomized NFS/*sld* mice as a mouse model for SjD. In the SjD mouse model, lymphocytic infiltration around the ducts of the salivary and lacrimal glands is observed, along with inflammatory lesions in the lungs [21]. Furthermore, anti‐SS‐A and anti‐SS‐B autoantibodies are detected in the serum of this model [22], closely resembling the pathological and clinical features of patients with SjD.

In this study, we investigated the dynamics of ABCs in the target organ of SjD using the thymectomized NFS/*sld* mouse model as well as clinical samples from patients with SjD. Furthermore, we examined the potential contribution of ABCs to the formation of SjD‐related pathology. Our findings may offer new insights into the cellular mechanisms of age‐related disorders, including autoimmune diseases.

## 2. Materials and Methods

### 2.1. Human Subjects

The pathological specimens of labial SGs (LSGs) were obtained from biopsies for diagnostic purposes at Tokushima University Hospital. The study was approved by the Ethics Committee of Tokushima University Hospital (Number 4252‐2). The study used control (*n* = 11) and SjD patients (*n* = 44), and all participants gave written informed consent according to the principles outlined in the Declaration of Helsinki. All SjD patients were diagnosed according to the Ministry of Health SjD Research Committee (1999) and the American College of Rheumatology classification (2016). Male patients were excluded from this cohort. Clinical information is presented in Supporting Information 1: Table S1 and Supporting Information 2: Table S2.

### 2.2. Animals

NFS/*sld* mice were obtained from the Central Institute for Experimental Animals (Kawasaki, Japan). This study was designed and undertaken by following the “Fundamental Guidelines for Proper Conduct of Animal Experiments and Related Activities in Academic Research Institutions” under the jurisdiction of the Ministry of Education, Culture, Sports, Science, and Technology of Japan. The protocol was approved by the Committee on Animal Experiments of Tokushima University (Permit Numbers T2022‐70 and T2024‐6) and the Institutional Animal Care and Use Committee of Institute of Science Tokyo (Approval Number A2024‐134A). The thymus gland of 3‐day‐old NFS/*sld* mice was removed under anesthesia to establish a SjD model mouse [21–24]. All mice used in this study were female. Nonthymectomized female NFS/*sld* mice were used as controls. Among control and SjD model mice, young was defined as 6–8 weeks of age (control; *n* = 6–8 and SjD model; *n* = 7–8), mature‐adult was defined as 12–25 weeks of age (control; *n* = 3–8 and SjD model; *n* = 4–9), and middle‐aged was defined as 48 weeks of age (control; *n* = 4 and SjD model; *n* = 4) based on guideline [25]. In addition, among SjD model mice, the predisease stage was defined as 6 weeks of age, the onset stage was defined as 8 weeks of age, and the disease stage was defined as 12–48 weeks of age.

### 2.3. Histological Evaluation

SG tissues were fixed with 10% phosphate‐buffered formaldehyde (pH 7.2) and prepared for histological examination. Sections were stained with hematoxylin and eosin (HE). The pathological grade was quantified by counting the number of lymphocytic foci per unilateral lobe of SGs using NIS‐Elements D software (Nikon Corporation).

### 2.4. Immunofluorescence Analysis for Human Subjects

Formalin‐fixed paraffin‐embedded (FFPE) sections of LSG tissues from non‐SjD sicca and SjD patients were used; antigen retrieval by heat was performed in ImmunoActive (Matsunami Glass Industries, Inc.), and the sections were incubated in Blocking One Histo (nacalai tesque). The sections were then incubated with antihuman CD20 (L26, Cell Signaling Technology) and antihuman T‐bet (E412K, Cell Signaling Technology) antibodies for 1 h at room temperature. After washing with PBS, sections were incubated with Alexa Fluor 488‐conjugated anti‐mouse IgG (Invitrogen) and Alexa Fluor 546‐conjugated anti‐rabbit IgG (Invitrogen) as secondary antibodies at room temperature for 1 h. To determine whether T‐bet‐expressing cells were B cells or CD4^+^ T cells in human tissues, rabbit antihuman CD4 antibody (ST0488; Invitrogen) was labeled using the FlexAble CoraLite Plus 647 Antibody Labeling Kit for Rabbit IgG (Proteintech). Following antigen retrieval and blocking, sections were incubated with mouse antihuman CD20 (L26; Cell Signaling Technology) and rabbit antihuman T‑bet (E412K; Cell Signaling Technology) primary antibodies for 1 h at room temperature. After washing with PBS, sections were incubated with Alexa Fluor 488‐conjugated anti‑mouse IgG and Alexa Fluor 546‐conjugated anti‑rabbit IgG (both Invitrogen) as secondary antibodies for 1 h at room temperature. To remove unbound antibodies, samples were washed five times with PBS for 3 min each, and then incubated with a custom Alexa Fluor 647–‐conjugated antihuman CD4 antibody for 1 h at room temperature. The sections were then stained with 4′, 6‐diamidino‐2‐phenylindole (DAPI, Invitrogen) and observed under a confocal microscope (Nikon A1R+, Nikon).

### 2.5. Single Cell Isolation

To isolate single cells from spleen (SP) and cervical lymph nodes (cLNs) of control and SjD model mice, these tissues were homogenized using gentle MACS Dissociators (Miltenyi Biotec) in Roswell Park Memorial Institute (RPMI)‐1640 containing 10% FBS. The cells were then passed through a 70 µm filter, and after centrifugation, 9 mL of NH_4_Cl and 1 mL of Tris buffer were added and allowed to react for 10 min. To isolate single cells from the SGs, SGs were digested with collagenase D (Roche) and DNase I (Roche) in 10% FBS‐supplemented Dulbecco’s modified Eagle’s medium (DMEM) at 37°C for 35–40 min with a MACS tissue dissociator (Miltenyi Biotec).

### 2.6. Flow Cytometric Analysis

Single cells from SGs, cLNs, and SP of control and SjD model mice were incubated with FITC‐conjugated anti‐mouse CD11c (N418, BioLegend), PE‐conjugated anti‐mouse CD95 (15A7, Invitrogen), PECy7‐conjugated anti‐mouse CD19 (6D5, BioLegend), Brilliant Violet 510‐conjugated anti‐mouse CD45 (30F11, BioLegend), and Pacific Blue‐conjugated anti‐mouse CD11b (M1/70, BioLegend) antibodies in the dark at 4°C for 30 min. For intracellular staining, Foxp3 Fixation/Permeabilization working solution (Invitrogen) was added, and the cells were reacted in the dark for 30 min at room temperature, washed with 1 × permeabilization buffer, centrifuged, and then stained with APC‐conjugated anti‐mouse/human T‐bet (4B10, BioLegend) antibody for 30 min in the dark at room temperature. The gating strategy for detecting ABCs was shown in Supporting Information 3: Figure S1. Data were collected on a CytoFLEX S (Beckman Coulter) and analyzed using FlowJo software (BD Biosciences).

### 2.7. Saliva Flow Evaluation

For saliva measurement, the mice were anesthetized with ketamine (60 mg/kg body weight) and xylazine (6 mg/kg body weight) and orally administered pilocarpine (0.5 mg/kg body weight). Saliva volume was evaluated by measuring the weight of the filter paper after the saliva was absorbed for 20 min after pilocarpine stimulation.

### 2.8. In Vitro ABC Differentiation Assay

Purified B cells were obtained using the EasySep Mouse B Cell Isolation Kit (STEMCELL Technologies) and stimulated with or without purified F(ab’)_2_ goat anti‐mouse IgM (μ chain) antibody (10 μg/mL, Poly21571, BioLegend), recombinant mouse CD40L (500 ng/mL, BioLegend), IL‐21 (50 ng/mL, BioLegend), and IFN‐γ (50 ng/mL, BioLegend). After 3 days of culture, the stimulated B cells were stained with APC‐conjugated anti‐mouse/human T‐bet antibody (4B10, BioLegend) and analyzed by flow cytometry.

### 2.9. Enzyme‐Linked Immunosorbent Assay (ELISA)

The concentrations of IL‐21 and IFN‐γ in the culture supernatants of B cells were measured using a Mouse IL‐21 ELISA Kit (ProteinTech) and a Mouse IFN‐γ ELISA Kit (ProteinTech), respectively. Briefly, B cells were isolated from the SP of mature‐adult control and SjD model mice using the EasySep Mouse B Cell Isolation Kit (STEMCELL Technologies) and cultured for 3 days in the presence of purified F(ab’)_2_ goat anti‐mouse IgM (μ chain) antibody (10 μg/mL, Poly21571, BioLegend) and recombinant mouse CD40L (500 ng/mL, BioLegend). After 3 days of culture, culture supernatants were collected and analyzed according to the manufacturers’ instructions.

### 2.10. RNA In Situ Hybridization (RNA‐ISH) With Immunofluorescence Analysis

For RNA‐ISH using FFPE sections of mouse SGs, the RNAscope Multiplex Fluorescent Reagent Kit v2 (Advanced Cell Diagnostics, Inc.) was used. For detection of each gene expression, RNAscope Probe‐Mm‐*Interleukin-21* (*Il21*)‐C1 (Advanced Cell Diagnostics, Inc.) and Opal 570 Reagent Product (Akoya Biosciences) were added. After hybridization of this probe, anti‐mouse CD4 (D7D2Z, Cell Signaling Technology) antibody was stained at room temperature for 1 h, and Alexa Fluor 488‐conjugated anti‐rabbit IgG (Invitrogen) was used as a secondary antibody. DNA was visualized by DAPI, Dojindo staining. Finally, stained sections were incubated with fluorescent mounting medium (Agilent Technologies). The fluorescence of these stained sections was observed with a confocal laser microscope (Nikon A1R+, Nikon). To evaluate what cell type the major expressor was, five or more *Il21* transcripts‐expressing cells were defined as *Il21* highly expressing (*Il21* ^high^) cells.

### 2.11. Cytokine Detection Assay

Mononuclear cell suspensions from SGs were stimulated with phorbol 12‐myristate 13‐acetate (PMA, 50 ng/mL, Sigma Aldrich) and ionomycin (1 µg/mL, Sigma Aldrich) in the presence of monensin (eBioscience) for 6 h. After incubation, these SG cells were stained with PE‐conjugated anti‐mouse CD4 (RM4‐5, BioLegend), Pacific Blue‐conjugated anti‐mouse CD11b (M1/70, BioLegend), PECy7‐conjugated anti‐mouse CD19 (6D5, BioLegend), and incubated with Amcyan‐conjugated anti‐mouse CD8α (53‐6.7, BioLegend) antibody for IFN‐γ detection. FITC‐conjugated anti‐mouse CD4 (RM4‐5, BioLegend), PECy7‐conjugated anti‐mouse CXCR5 (L138D7, BioLegend), and Brilliant Violet 510‐conjugated anti‐mouse CD45 (30F11, BioLegend) were used for IL‐21 detection. For intracellular staining, the Foxp3/Transcription Factor Staining Buffer Set (Invitrogen) was used. The permeabilization working solution was added to the suspension cells, and the cells were reacted in the dark for 30 min at room temperature, washed with 1 × permeabilization buffer, centrifuged, and then stained with FITC‐conjugated anti‐mouse IFN‐γ (XMG1.2, BioLegend), APC‐conjugated anti‐mouse/human T‐bet (4B10, BioLegend), and PE‐conjugated anti‐mouse IL‐21 (mhalx21, eBioscience) antibodies for 30 min in the dark at room temperature. Data were collected on a CytoFLEX S (Beckman Coulter) or FACS Canto II (BD Biosciences) and analyzed using FlowJo software (BD Biosciences).

### 2.12. Quantitative Reverse Transcription‐Polymerase Chain Reaction (qRT‐PCR)

Total RNA from the SGs was extracted with RNAiso Plus (TaKaRa Bio, Inc.) according to the manufacturer’s instructions. Total RNA was then reverse‐transcribed into cDNA by ReverTra Ace quantitative PCR RT Master Mix with gDNA Remover (TOYOBO). The target genes and *Gapdh* in the tissues were amplified with a StepOne Real‐Time PCR System (Applied Biosystems) using Fast SYBR Green Master Mix (Thermo Fisher Scientific) and the following primers: *Ifng*: forward, 5′‐ AGCGGCTGACTGAACTCAGATTGTAG‐3′; reverse, 5′‐ GTCACAGTTTTCAGCTGTATAGGG‐3′. *Gapdh*: forward, 5′‐ACTCCACTCACGGCAAATTC‐3′; reverse, 5′‐TCTCCATGGTGGTGAAGACA‐3′.

### 2.13. Statistics

These results are expressed as the mean ± standard error of the means (SEMs). One‐way ANOVA was performed for multiple comparisons, whereas Student’s *t*‐test was used for comparisons between two groups.

## 3. Results

### 3.1. ABCs in SGs of Patients With SjD

To determine whether ABCs infiltrate the SGs in SjD, we performed histological analysis using LSG sections from 44 patients with SjD and 11 patients with non‐SjD sicca symptoms (Supporting Information 1: Table S1A,B and Supporting Information 2: Table S2). ABCs are defined by the expression of the transcription factor T‐bet in B cells [8, 9, 26]. CD20^+^ T‐bet^+^ ABCs were detected within lymphocytic foci in the LSGs of SjD patients but were rarely observed in those from non‐SjD sicca patients (Figure 1A). In addition, to determine whether T‐bet‐expressing cells were B cells or CD4^+^ T cells, we performed histological analysis of SG sections from Grade 4 SjD patients. CD4^+^ T‐bet^+^ T cells were distinct from CD20^+^ T‐bet^+^ ABCs, indicating that ABCs are separate from Th1 cells within the same region of interest (Figure 1B,C). The number of ABCs per a lymphocytic focus was significantly higher in SjD patients than in non‐SjD sicca controls (Figure 1D). Previous studies have suggested that ABCs expand with aging [8, 9, 27]. Consistently, CD20^+^ T‐bet^+^ ABCs were rarely detected in SjD patients in their 20s–30s, whereas their numbers increased in patients in their 40s–60s compared to both younger (20s–30s) and older (70s–80s) patients (Figure 1E). In contrast, no correlation was observed between focus score and the number of ABCs (Figure 1F). These findings indicate that the number of ABCs in the LSGs of SjD patients increases after the age of 40, independently of focus score.

Figure 1Increased age‐associated B cells in minor salivary glands of patients with SjD. (A) Immunofluorescence analysis using labial salivary gland sections of non‐SjD sicca and SjD patients. DAPI (blue), CD20 (green), and T‐bet (red). (B) The number of ABCs per focus in LSG of non‐SjD sicca (black, *n* = 11) and SjD (red, *n* = 44) patients. (C) Merged image of immunofluorescence analysis using a labial salivary gland (LSG) section of a Greenspan Grade 4‐SjD patient. DAPI (white), CD20 (magenta), T‐bet (turquoise), and CD4 (green). (D) Age‐associated B cells (ABCs) were defined as T‐bet^+^ CD20^+^, and shown by red arrowhead (upper panels). Type‐1 helper T cells (Th1s) were defined as T‐bet^+^ CD4^+^, and shown by orange arrowhead (lower panels). (E) The number of ABCs per focus in SjD patients by each age layer. The numbers of patients in each age group were indicated below: 20–29 (*n* = 3), 30–39 (*n* = 4), 40–49 (*n* = 5), 50–59 (*n* = 9), 60–69 (*n* = 10), 70–79 (*n* = 9), and 80–89 (*n* = 4). (F) The number of ABCs per focus in SjD patients by each pathological focus score. The numbers of patients in each group were indicated below: Focus score = 1 (*n* = 5), 2 (*n* = 13), 3 (*n* = 8), 4 (*n* = 10), 5 (*n* = 4), 6 (*n* = 1), and 7 (*n* = 3). Data are shown as average ± SEM of human subjects.  ^∗∗∗^ *p* < 0.0005 (Student’s *t*‐test). Scale bar = 50 μm (A and C) and 10 μm (D).

**Figure:**
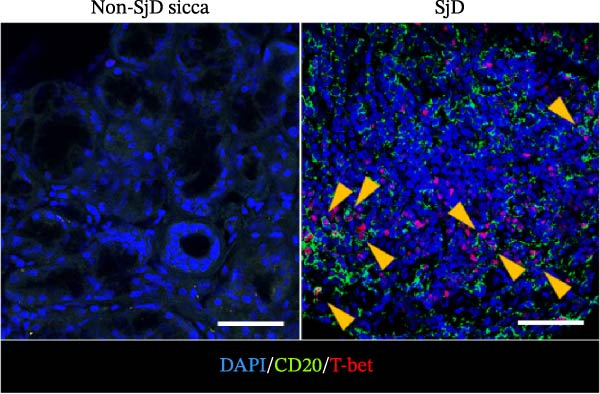
(A)

**Figure:**
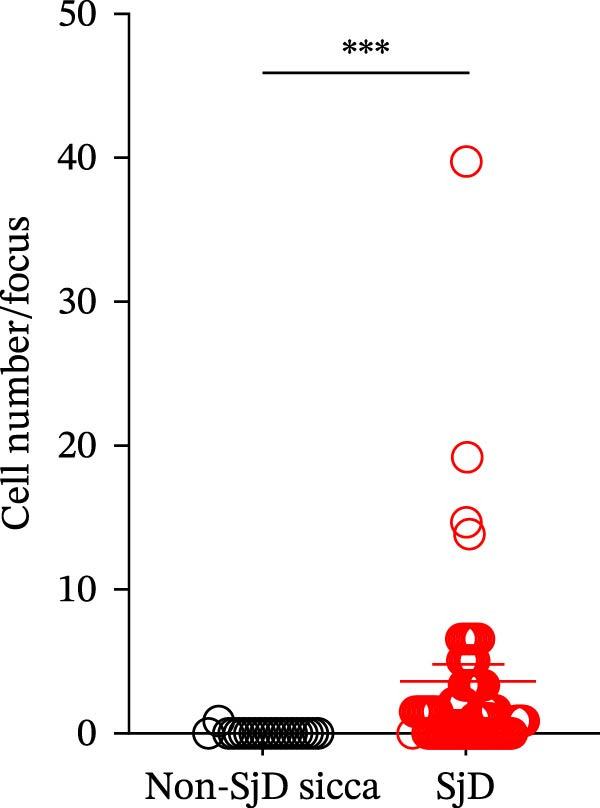
(B)

**Figure:**
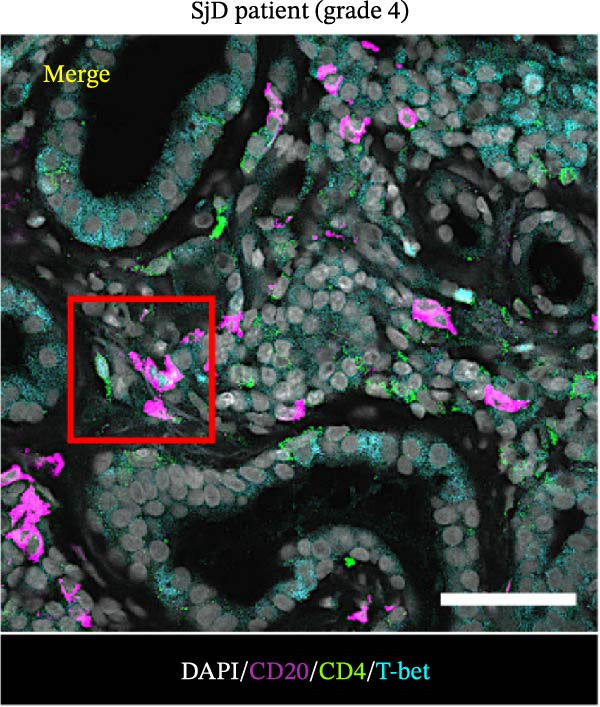
(C)

**Figure:**
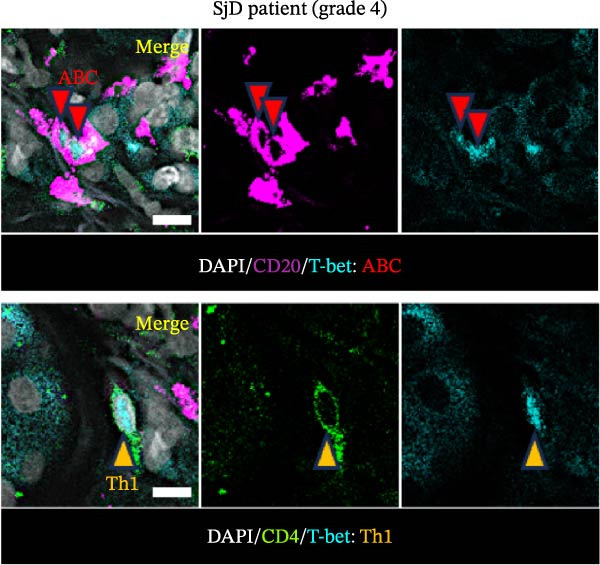
(D)

**Figure:**
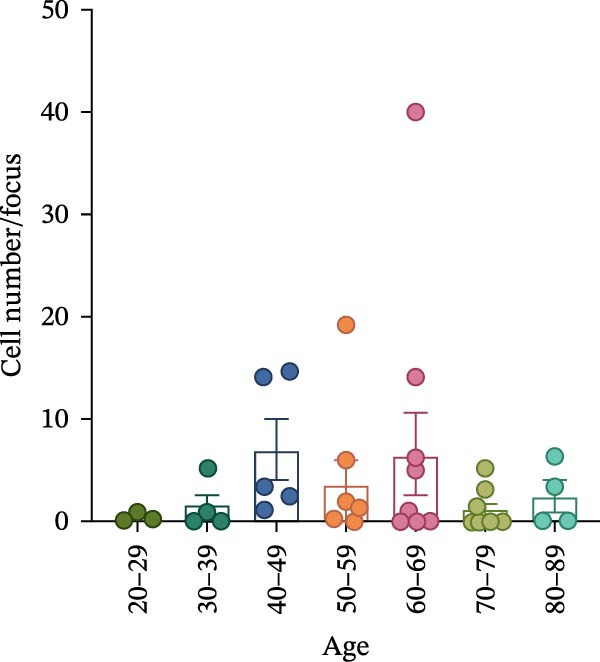
(E)

**Figure:**
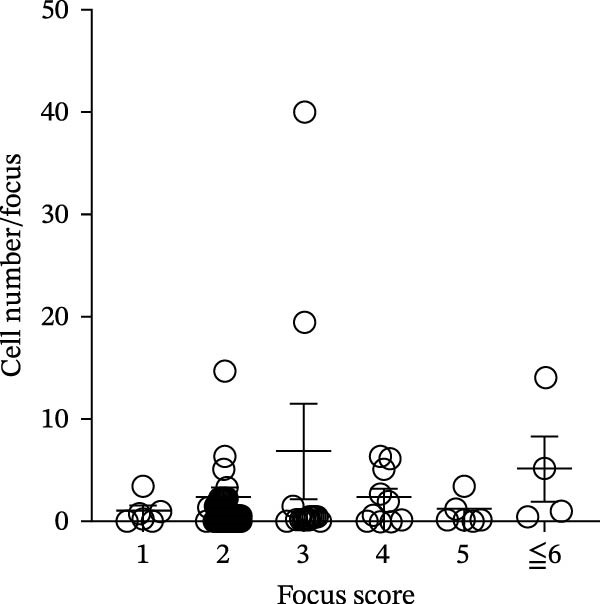
(F)

### 3.2. CD11b^+^ CD95^+^ ABCs in SGs of SjD Model Mice

To investigate the expansion of ABCs in the SGs, we used a SjD mouse model in which thymectomy was performed on Day 3 after birth [21–24]. In this SjD model, lymphocytic infiltration and impaired saliva secretion are observed beginning at 8 weeks of age [21–24], and infiltration in the SGs progressively increases with age up to 20 months [28]. Based on a previous study [25], we defined 6–8 weeks as the young phase, 16–25 weeks as the mature‐adult phase, and 48 weeks as the middle‐aged phase. In SjD model mice, the level of lymphocyte infiltration increased with age. Interestingly, in control mice, the number of lymphocytic foci was significantly higher during the middle‐aged phase compared with the young and mature‐adult phases. Therefore, no differences in the level of lymphocyte infiltration were observed between control and SjD model mice during the middle‐aged phase (Figure 2A,B). Similarly, flow cytometric analysis revealed no differences in the numbers of CD4^+^ T cells, CD8^+^ T cells, or B cells in the SGs between control and SjD model mice during this phase (Supporting Information 4: Figure S2A,B). These results indicate that while SjD‐like lesions appeared during the middle‐aged phase in sham‐operated NFS/*sld* control mice, thymectomy induced the early onset of SjD‐like pathology beginning in the mature‐adult phase in SjD model mice.

Figure 2ABCs in SGs of SjD model mice with aging (A) hematoxylin and eosin staining using SG sections from control (left) and SjD model (right) mice at young (upper), mature‐adult (middle), and middle‐aged (lower) phases. (B) The number of lymphocytic foci per lobe of SG from control (black) and SjD model (red) mice at each phase The numbers of mice in each group were indicated below: young (control; *n* = 8 and SjD model; *n* = 8), mature‐adult (control; *n* = 8 and SjD model; *n* = 8) and middle‐aged (control; *n* = 4 and SjD model; *n* = 4). (C) Flow cytometric panels using SG cells from control (left) and SjD model (right) mice at young (upper), mature‐adult (middle), middle‐aged (lower) phase. All panels were gated on CD19^+^ CD45^+^ cells. ABCs were defined as CD11*b* ^+^ CD95^+^ CD19^+^ CD45^+^ cells. (D) The number of ABCs in SGs from control (black) and SjD model (red) mice at each phase. The numbers of mice in each group were indicated below: young (control; *n* = 6 and SjD model; *n* = 7), mature‐adult (control; *n* = 7 and SjD model; *n* = 7) and middle‐aged (control; *n* = 4 and SjD model; *n* = 4). These results shown in this figure are representative data from at least four experiments and are expressed as the mean ± SEMs.  ^∗^ *p* < 0.05,  ^∗∗^ *p* < 0.005 (one‐way ANOVA). Scale bar = 100 μm (A).

**Figure:**
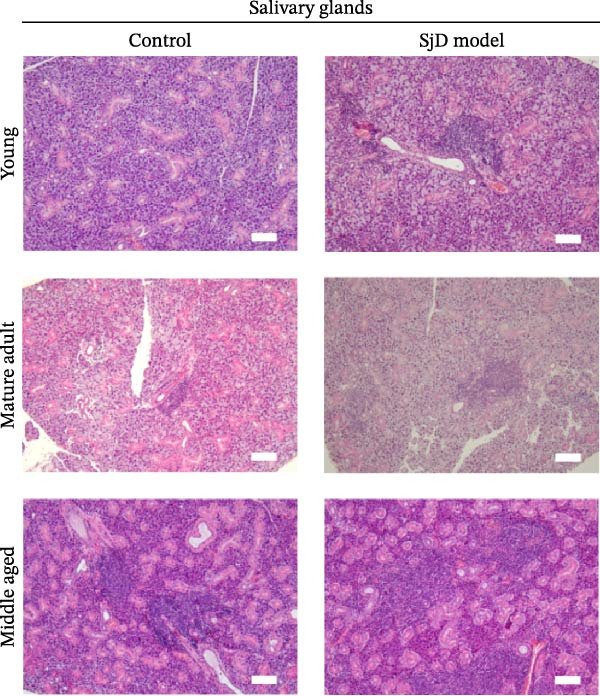
(A)

**Figure:**
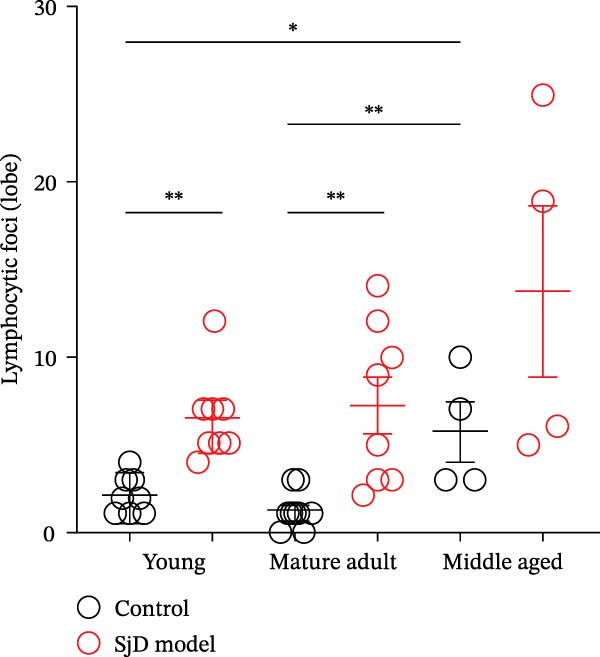
(B)

**Figure:**
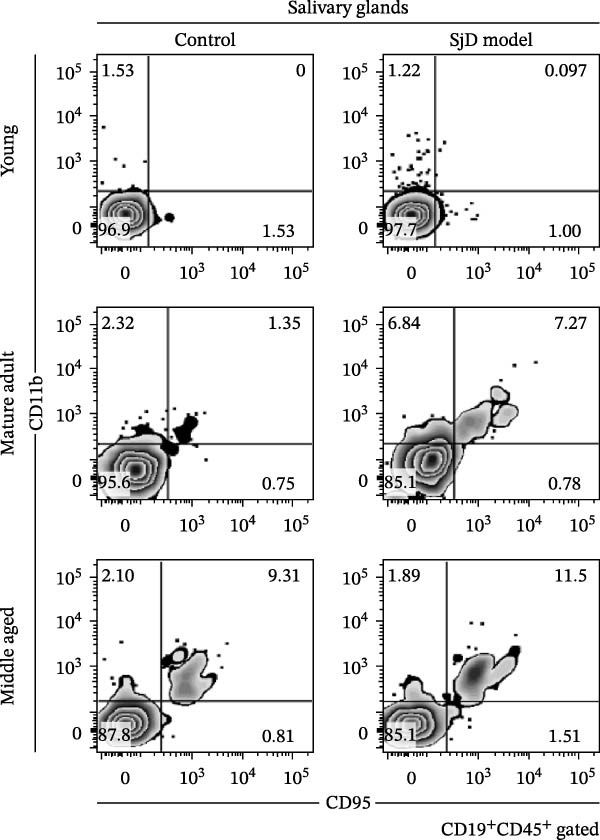
(C)

**Figure:**
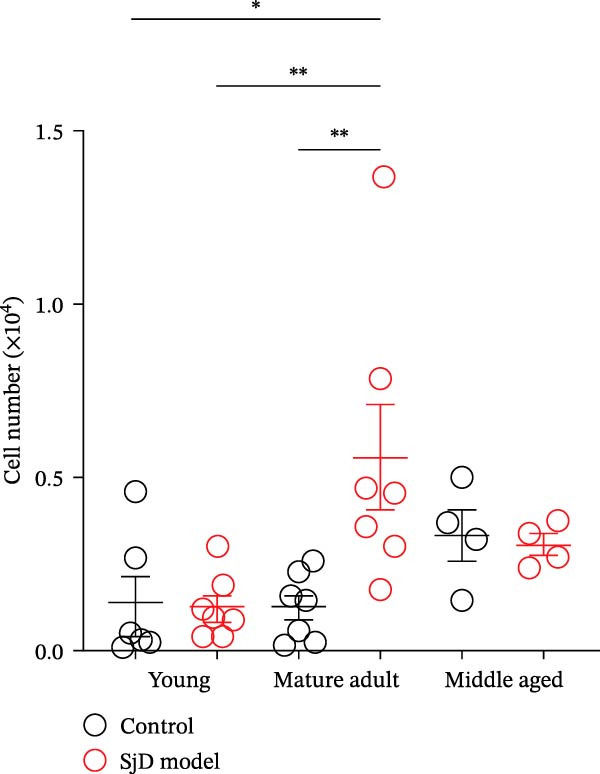
(D)

Based on previous studies, ABCs are phenotypically defined as CD11*b* ^+^ CD95^+^ CD19^+^ CD45^+^ cells (Supporting Information 3: Figure S1A) [8–11, 14, 17, 18]. In addition, intracellular expression of the transcription factor T‐bet, a hallmark of ABCs, was higher in CD11*b* ^+^ CD95^+^ B cells compared to CD11*b* ^+^ CD95^−^ and CD11*b* ^−^ CD95^−^ B cells (Supporting Information 3: Figure S1B,C). During the young phase, there was no significant difference in the number of ABCs in SGs between control and SjD model mice (Figure 2C,D). However, in the mature‐adult phase, ABCs were significantly increased in the SGs of SjD model mice compared to controls. Moreover, the number of SG‐ABCs in mature‐adult SjD mice was higher than that in young SjD mice (Figure 2C,D). By contrast, no significant difference in ABC numbers was observed between middle‐aged control and SjD model mice (Figure 2C,D). These results indicate that ABCs expand in the SGs of control mice during the middle‐aged phase. Moreover, in this SjD model, both SjD development and ABC expansion are accelerated, occurring earlier in the mature‐adult phase. To determine whether the accumulation of ABCs was driven by aging or occurred in association with disease onset, we compared the number of ABCs in the SGs across three stages: predisease, onset, and disease. In this SjD model, a reduction in salivary flow was observed from 8 weeks of age (Supporting Information 3: Figure S1D); therefore, mice at 6 weeks of age were defined as the predisease stage, those at 8 weeks of age as the onset stage, and those older than 8 weeks, in whom disease pathology was already established, were defined as the disease stage. The number of ABCs in the SGs was significantly increased in the disease stage compared with both the predisease and onset stages (Supporting Information 3: Figure S1E). These findings suggested that ABCs did not expand abruptly at disease onset but instead gradually accumulated with age.

### 3.3. Comparison of CD11b^+^ CD95^+^ ABCs Between SGs and Lymphoid Tissues of Mature‐Adult Phase

Previous studies have reported that ABCs increase in lymphoid tissues and contribute to the pathogenesis of autoimmune diseases [14–19, 29–31]. To investigate whether a similar pattern occurs in our model, we examined cLNs and SP from mature‐adult control and SjD model mice. A small population of CD11*b* ^+^ CD95^+^ ABCs was detected in the cLNs and SP of both control and SjD model mice (Figure 3A). However, the frequency and number of ABCs in the cLNs and SP did not differ between the two groups (Figure 3B). In contrast, when comparing SGs with cLNs and SP, the frequency of ABCs among B cells was significantly higher in SGs than in cLNs and SP of mature‐adult SjD model mice (Figure 3B). These findings suggest that ABC accumulation may be easier to occur in SG rather than lymphoid tissue, uniquely occurring in the target organ, in this case, the SGs, of SjD model mice during the mature‐adult phase.

Figure 3Expansion of ABCs within the SGs in SjD model mice. (A) Flow cytometric panels using SGs (upper), cervical lymph nodes (cLNs, middle), and spleen (SP, lower) from control (left) and SjD model (right) mice at the mature‐adult phase. All panels were gated on CD19^+^ CD45^+^ cells. ABCs were defined as CD11*b* ^+^ CD95^+^ CD19^+^ CD45^+^ cells. (B) The frequency of ABCs among CD19^+^ B cells in SGs, cLNs, and SP from control (black) and SjD model (red) mice at the mature‐adult phase. The numbers of mice in each group were indicated below: SG (control; *n* = 6 and SjD model; *n* = 6), cLNs (control; *n* = 6 and SjD model; *n* = 6), and SP (control; *n* = 5 and SjD model; *n* = 5). These results shown in this figure are representative data from at least four experiments and are expressed as the mean ± SEMs.  ^∗∗^ *p* < 0.005,  ^∗∗∗∗^ *p* < 0.00005 (one‐way ANOVA).

**Figure:**
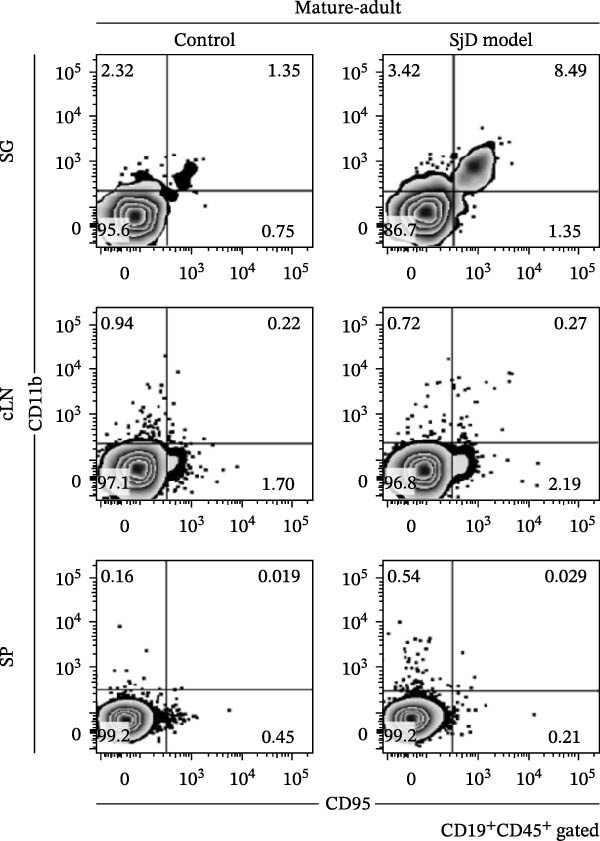
(A)

**Figure:**
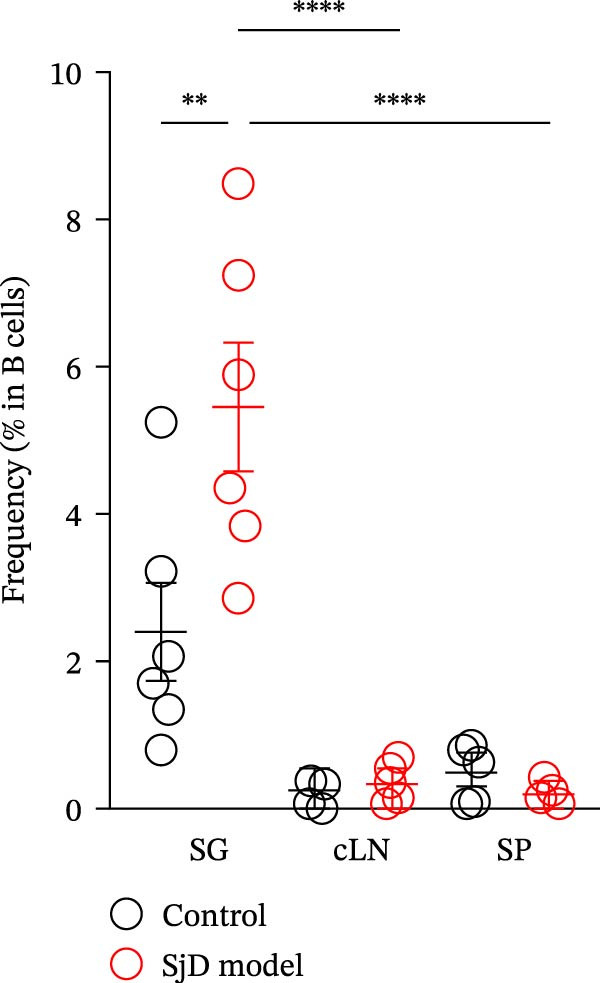
(B)

### 3.4. Requirement of IL‐21 and IFN‐γ on ABC Differentiation in Mature‐Adult SjD Model

Previous studies have already suggested that IL‐21 and IFN‐γ induce ABC differentiation [17–20]. To evaluate the requirement of IL‐21 and IFN‐γ on ABC differentiation, we performed in vitro induction of ABC differentiation. The purified splenic B cells from control and SjD model mice at the mature‐adult phase were stimulated with or without anti‐IgM antibody, recombinant CD40L, IL‐21, and IFN‐γ for 3 days (Figure 4A,B). The frequency and geometric mean fluorescence intensity (gMFI) of T‐bet were significantly elevated by the treatment of IL‐21 and IFN‐γ (Figure 4A,C,D). In addition, these levels of T‐bet were significantly higher in mature‐adult SjD model‐B cells compared to mature‐adult control‐B cells (Figure 4B,C,D). These findings suggested that IL‐21 and IFN‐γ are important for ABC differentiation, and mature‐adult SjD model‐B cells are easier to differentiate into ABCs.

Figure 4The effect of IL‐21 and IFN‐γ in mature‐adult SG on ABC differentiation. (A) The histogram of T‐bet expression gated on CD19^+^ B cells of control (left) and SjD model (right) with or without anti‐IgM antibody, recombinant mouse CD40L, IL‐21, and IFN‐γ. (B) The histogram of T‐bet expression gated on CD19^+^ B cells of control (gray) and SjD model (red) in the presence of anti‐IgM antibody, recombinant mouse CD40L, IL‐21, and IFN‐γ. (C) The frequency of T‐bet^+^ in B cells of control (black) and SjD model (red) with or without anti‐IgM antibody, recombinant mouse CD40L, IL‐21, and IFN‐γ. (D) The gMFI of T‐bet in B cells of control (black) and SjD model (red) with or without anti‐IgM antibody, recombinant mouse CD40L, IL‐21, and IFN‐γ. These results shown in this figure are representative data from at least four experiments and are expressed as the mean ± SEMs (*n* = 4).  ^∗∗^ *p* < 0.005,  ^∗∗∗∗^ *p* < 0.00005 (one‐way ANOVA).

**Figure:**
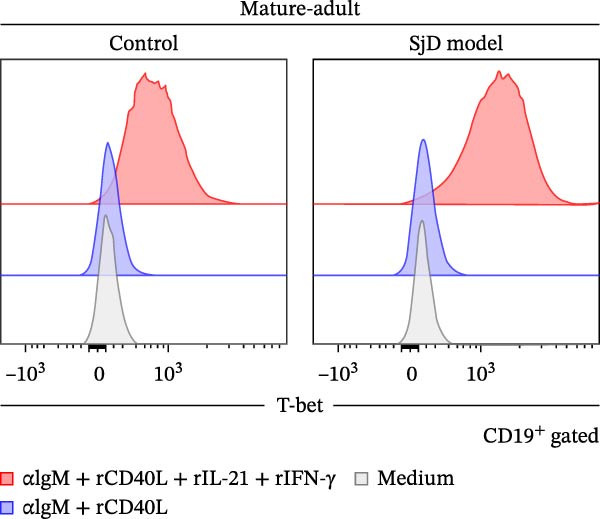
(A)

**Figure:**
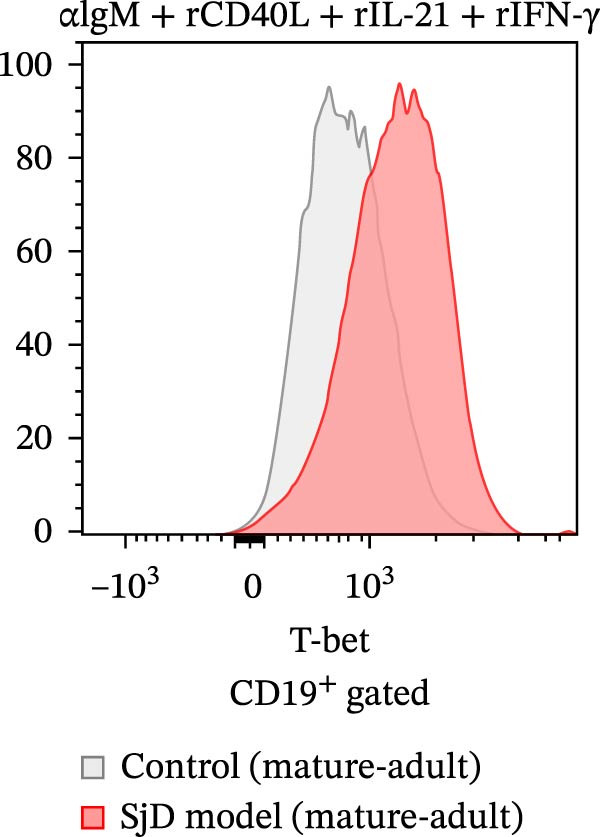
(B)

**Figure:**
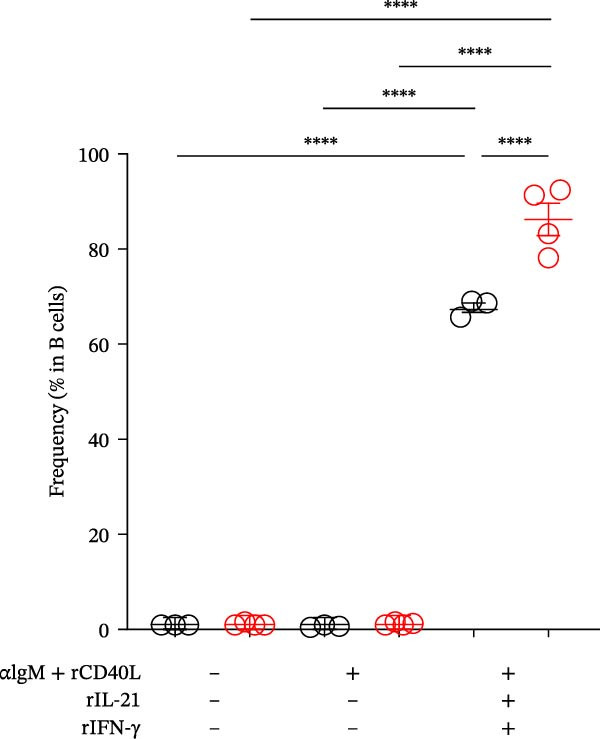
(C)

**Figure:**
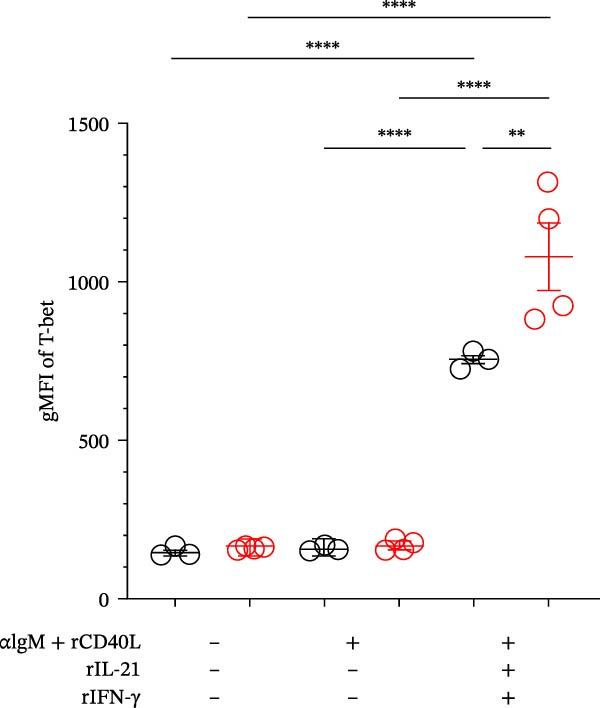
(D)

To determine whether the enhanced susceptibility of SjD model‐B cells to ABC differentiation was due to increased autocrine production of IL‐21 and IFN‐γ, we measured the concentrations of IL‐21 and IFN‐γ in the culture supernatants of B cells stimulated with anti‐IgM antibody and recombinant CD40L. No significant differences in the levels of IL‐21 or IFN‐γ were observed between control and SjD model B cells (Supporting Information 5: Figure S3A,B). These results suggested that ABC differentiation in SjD model B cells is not driven by increased cytokine production from B cells themselves but is more likely promoted by IL‐21 and IFN‐γ derived from other immune cell populations.

### 3.5. IL‐21 Produced From CD4^+^ T Cells in SGs of Mature‐Adult SjD Model Mice

CD4^+^ T cells have been shown to drive the differentiation of naïve B cells into ABCs through IL‐21 signaling [8–11]. To determine whether CD4^+^ T cells produce IL‐21 in the SGs, we performed a combined assay using ISH to detect *Il21* gene expression and immunofluorescence staining for the CD4 molecule in SG sections from young, mature‐adult, and middle‐aged control and SjD model mice. CD4^+^ T cells expressing the *Il21* gene were detected in the SGs of mature‐adult and middle‐aged SjD model. However, *Il21*‐expressing CD4^+^ cells were readily detectable in SG tissue of mature‐adult SjD model mice, whereas only a small number of IL‐21‐producing CD4^+^ cells were observed in middle‐aged SjD model mice. Similarly, *Il21*‐expressing CD4^+^ cells were rarely found in young mice of either group (Figure 5A). To quantify IL‐21‐producing cells, we defined *Il21* ^high^ cells as those expressing more than five *Il21* transcripts (Figure 5B) and counted these cells within each lymphocytic focus. The frequency of *Il21* ^high^ cells per focus was significantly higher among CD4^+^ T cells compared to CD4^-^ cells (Figure 5C). Among CD4^+^ T cell subsets, follicular helper T (Tfh) cells produced IL‐21 and helped B cell activation [32]. Another study suggested that Tfh cells were increased in our SjD model mice [22]. To confirm whether our IL‐21‐expressing CD4^+^ T cells were Tfh cells, we evaluated IL‐21 expression on Tfh cells by using CXCR5 as a Tfh cell marker. Then, the gMFI of IL‐21 was significantly higher in CXCR5^+^CD4^+^ T cells compared to CXCR5^−^ CD4^+^ T cells (Figure 5D,E). These findings suggest that IL‐21 production by Tfh cells may contribute to ABC differentiation in the SGs of mature‐adult SjD model mice.

Figure 5IL‐21 producing CD4^+^ T cells in SGs of SjD model mice. (A) In situ hybridization with immunofluorescence using SG sections of young (upper), mature‐adult (middle), and middle‐aged (lower), or control (left) and SjD model mice (right). DAPI (blue), CD4 (green), and *Il21* (red). (B) High magnification of mature‐adult SjD model mice section. *Il21* ^high^ was defined as > 5 transcripts of expression of *Il21*. *Il21* ^high^ CD4^+^ T cells were shown by arrowheads (orange). DAPI (blue), CD4 (green), and *Il21* (red). (C) The frequency of CD4^+^ or CD4^−^ cells among *Il21* ^high^ cells (*n* = 3). (D) FACS panels of CD4 and CXCR5 gated on CD4^+^ CD45^+^ SG cells (left) and histogram of IL‐21 gated among CXCR5^+^ (red) and CXCR5^−^ (blue) CD4^+^ T cells and IL‐21‐unstained control (gray) (right). (E) The gMFI of IL‐21 among CXCR5^+^ (red) and CXCR5^−^ (black) CD4^+^ T cells (*n* = 6). These results shown in this figure are representative data from at least four experiments and are expressed as the mean ± SEMs.  ^∗^ *p* < 0.05,  ^∗∗^ *p* < 0.005 (Student’s *t*‐test). Scale bar = 50 μm (A) and 10 μm (B).

**Figure:**
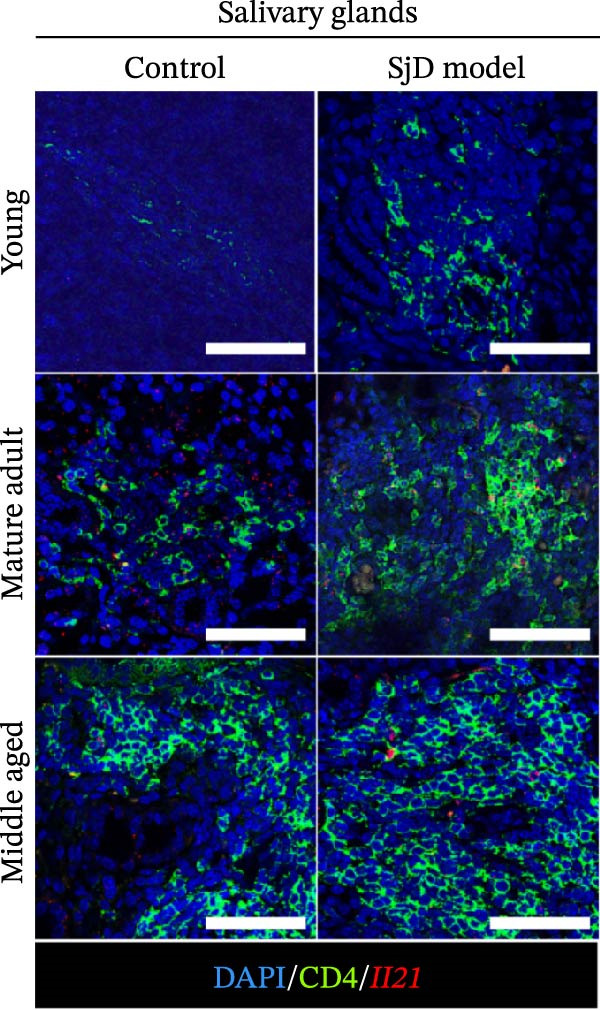
(A)

**Figure:**
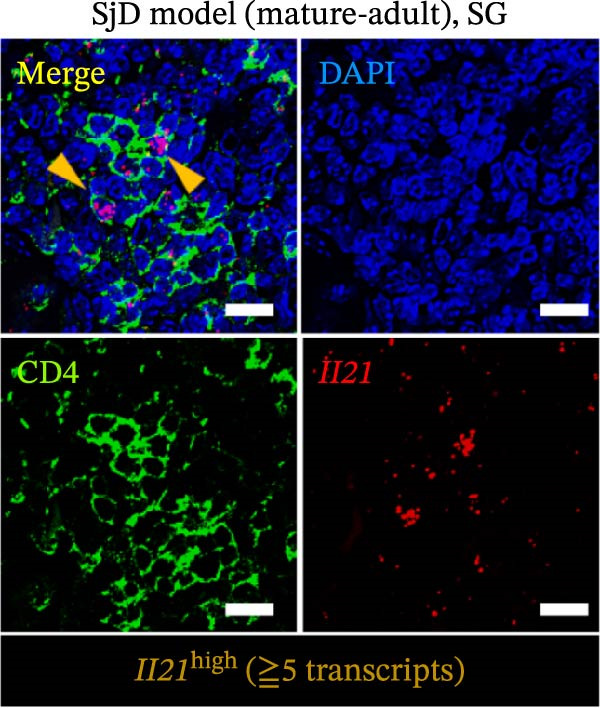
(B)

**Figure:**
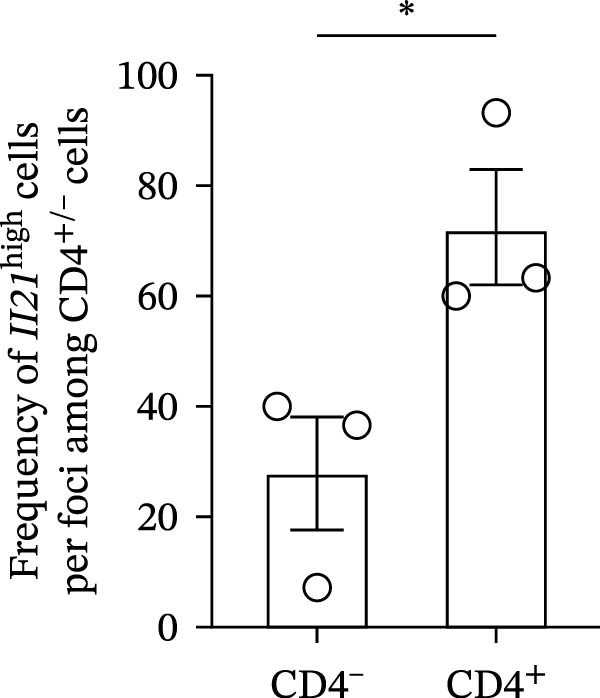
(C)

**Figure:**
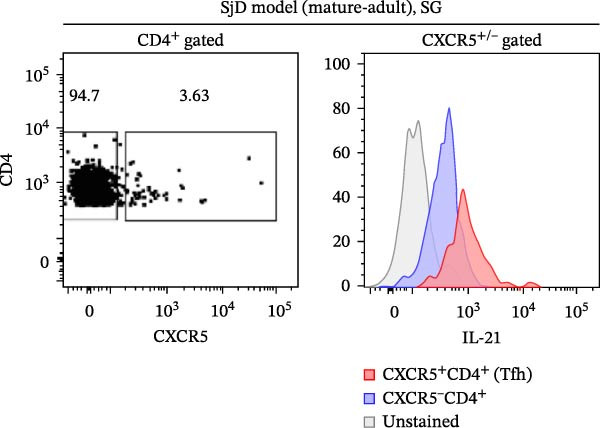
(D)

**Figure:**
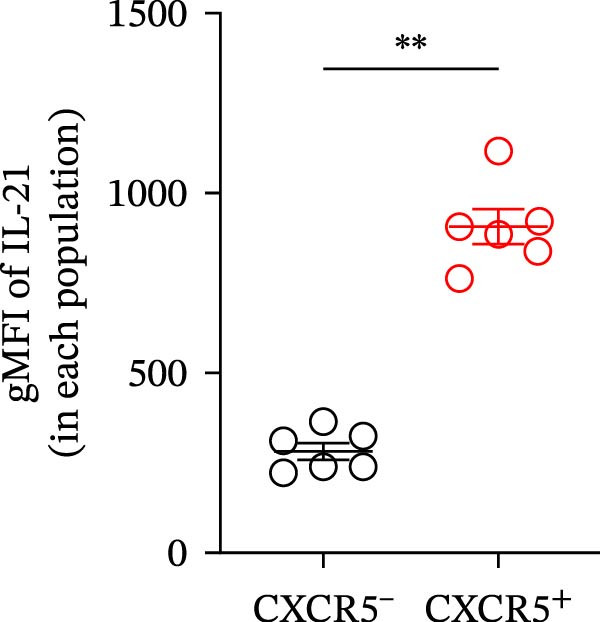
(E)

### 3.6. IFN‐γ Production for ABCs in SGs of Mature‐Adult SjD Model Mice

IFN‐γ is also known to be important for ABC differentiation [8–11]. Moreover, ABCs have been reported to contribute to proinflammatory responses through the secretion of IFN‐γ [12]. To identify which immune cell types produce IFN‐γ in the target tissue of SjD model mice, we performed flow cytometric analysis on various immune cell populations isolated from the SGs of mature‐adult SjD model mice. Intracellular expression of IFN‐γ was significantly higher in ABCs compared to conventional B cells, monocytes, Th1 cells, non‐Th1 CD4^+^ T cells, and CD8^+^ T cells (Figure 6A,B). These results suggest that robust IFN‐γ production by ABCs may not only contribute to local inflammation but also promote ABC expansion and differentiation through an autocrine mechanism, thereby exacerbating the autoimmune response in the SGs of mature‐adult SjD model mice. In addition, *Ifng* mRNA expression was significantly higher in SGs compared with cLNs in SjD model mice (Figure 6C). These findings suggest the possibility of tissue‐specific ABC expansion within the SGs of SjD model mice.

Figure 6IFN‐γ secretion by ABCs within SGs in SjD model mice. (A) Intracellular IFN‐γ expression of several immune populations using SGs from mature‐adult SjD model mice. Each immune population was defined as following expression pattern: ABCs; T‐bet^+^ CD11*b* ^+^ CD19^+^ CD45^+^, Bconv (conventional B cells); T‐bet^-^ CD11*b* ^−^ CD19^+^ CD45^+^, Monocytes; CD11*b* ^+^ CD19^−^ CD45^+^, Th1 (type 1 helper T cells); T‐bet^+^ CD4^+^ CD45^+^, non‐Th1; T‐bet^−^ CD4^+^ CD45^+^, CD8 (CD8^+^ T cells); CD8α^+^ CD45^+^. (B) The geometric mean fluorescence intensity (gMFI) of IFN‐γ in each immune population (*n* = 9). (C) *Ifng* mRNA expression level of cLNs and SGs from mature‐adult SjD model mice. The value of SGs was normalized by that of cLNs (*n* = 6). These results shown in this figure are representative data from at least four experiments and are expressed as the mean ± SEMs.  ^∗^ *p* < 0.05,  ^∗∗∗^ *p* < 0.0005,  ^∗∗∗∗^ *p* < 0.00005 (B: one‐way ANOVA and C: Student’s *t*‐test).

**Figure:**
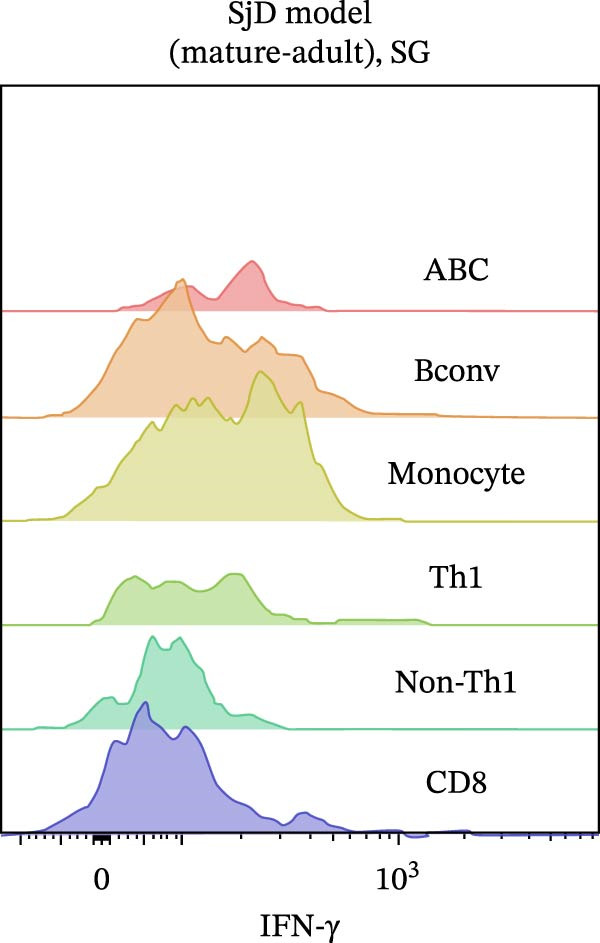
(A)

**Figure:**
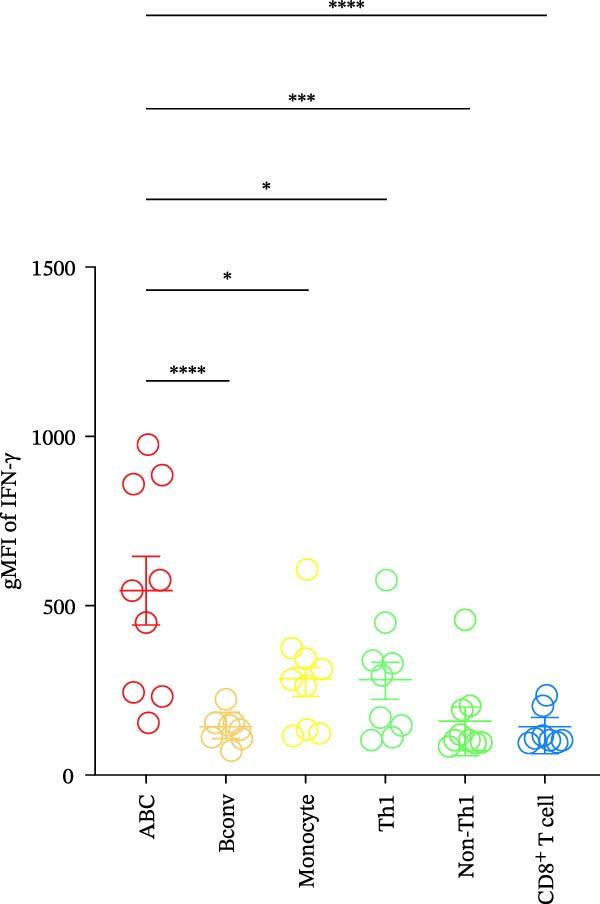
(B)

**Figure:**
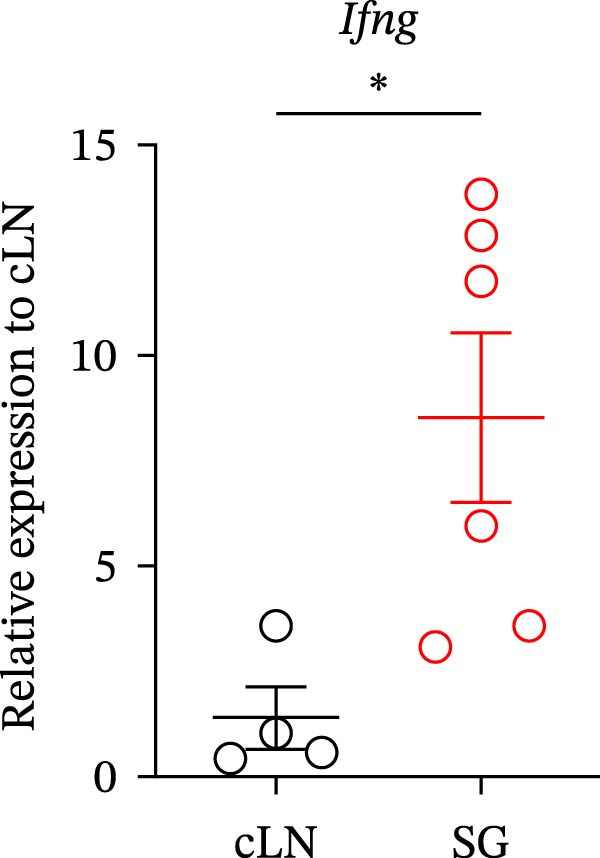
(C)

## 4. Discussion

To investigate the dynamics and functional roles of ABCs in the pathogenesis of SjD, we employed the thymectomized NFS/*sld* mouse model. This model is thought to develop SjD‐like lesions because of immune dysregulation centered on CD4^+^ T cells. Previous studies have reported reductions in both thymus‐derived and peripherally induced regulatory T (Treg) cells [33], impaired immunosuppressive function of Treg cells [34], and abnormal expansion of Tfh cells accompanied by enhanced B‐cell activation [22]. Consistent with these findings, prominent infiltration of CD4^+^ T cells and B cells is observed around the ductal epithelium of the SGs [22, 35]. However, the detailed phenotypic characteristics of B cells within the SGs have not been fully elucidated. In this SjD model, increased apoptosis within SG tissue and elevated levels of the autoantigen α‐fodrin have been reported at ~20 weeks of age [28], yet the temporal evolution of immune cell populations leading up to this stage has remained unclear. In the present study, we focused on ABCs, a B‐cell subset increasingly recognized for its role in autoimmune diseases, and characterized their dynamics across defined age‐based stages.

In the neonatal thymectomy model, alterations in T cell‐dependent immune responses mediated by Th cells, Treg, and Tfh cells can be analyzed through comparison with clinical data obtained from the patients. Furthermore, because T cell dysfunction represents a primary event in this model, secondary abnormalities in non‐T cell immune populations, including macrophages, natural killer cells, and B cells, can also be investigated within this framework [22, 24, 33–36]. In contrast, abnormalities in T cell‐independent immune responses, particularly those initiated by innate immune mechanisms, may be difficult to adequately assess using this model. SjD is characterized by complex interactions among multiple immune cell populations, including but not limited to T cells, as well as genetic and environmental factors that collectively shape disease pathogenesis. It is difficult to explain the pathogenesis of SjD solely on the basis of thymic abnormalities. Therefore, analyses based solely on the neonatal thymectomy model may not fully recapitulate the multifactorial nature of the disease.

At the young stage, histopathological scores in the SGs were significantly increased in SjD model mice compared with controls, whereas no significant change in ABC numbers was observed. Moreover, the lack of difference in SG ABC numbers between the predisease stage (6 weeks of age) and the onset stage (8 weeks of age) suggests that ABCs do not expand abruptly at disease onset. During the mature‐adult stage, SG pathology remained significantly elevated in SjD model mice, and ABCs selectively accumulated in the SGs, while remaining scarce in secondary lymphoid tissues. Upon progression to the middle‐aged stage, control mice also exhibited increased histopathological scores in the SGs, accompanied by a concomitant increase in ABCs. Collectively, these findings suggest that ABC expansion is not an immediate consequence of disease onset but rather occurs gradually after disease initiation in association with persistent CD4^+^ T‐cell dysregulation.

ABCs are a distinct effector B cell subset that expands with aging, infections, and autoimmune diseases [8–11]. In mice, this population is generally characterized as CD11*c* ^+^ CD11*b* ^+^ CD21^−^ CD23^−^ T‐bet^+^, while in humans, ABCs are defined as CD11*b* ^+^ CD11*c* ^+^ CD95^+^ CD21^−^ CD27^−^ CXCR5^−^ FCRL5^+^ IgD^−^ T‐bet^+^ [19]. In our study, we identified ABCs as T‐bet^+^ CD20^+^ cells in patients with SjD and as CD11*b* ^+^ CD95^+^ CD19^+^ cells in SjD model mice. Previous reports have shown that ABCs are elevated in the peripheral blood mononuclear cells of SjD patients compared to healthy controls [29–31]. Another study reported the infiltration of CD11*c* ^+^ ABCs into the parotid glands of SjD patients [37]. Our current analysis, which examined LSG samples from 55 individuals, including both non‐SjD sicca and SjD patients, is clinically significant for understanding the role of ABCs in SjD pathogenesis. However, it remains unclear whether the observed findings reflect mechanisms analogous to those identified in mouse models. Given the fundamental differences in aging processes between humans and mice—particularly with respect to sex hormone regulation and reproductive function [38]—direct age‐based comparisons are inherently limited. Although disease duration is an important factor for understanding disease chronicity when evaluating patient biopsy samples, it varied widely among individuals in the present study and could not be reliably determined from biopsy specimens alone. Therefore, age stratification was used as a surrogate analytical framework. Consequently, the relationship between age and disease duration could not be clearly defined, and age‐dependent changes in ABC accumulation in SjD patients could not be definitively established.

Splenic ABCs have been shown to increase with aging in many previous studies [14–19]. It has also been demonstrated that ABCs are elevated in the SGs of aged C57BL/6 mice (older than 1 year) compared to younger mice, a phenomenon attributed to the age‐associated increase in Tfh cells [39]. However, the mechanisms underlying ABC differentiation within the SGs remain unclear. Recent studies have begun to clarify the pathogenic roles of ABCs in autoimmune disease models. For instance, single‐cell RNA sequencing revealed the heterogeneity of ABCs in MRL/*lpr* mice, a one of lupus model [14]. In NOD.B10 mice, a one of SjD model, ABCs were shown to contribute to autoantibody production through TLR7 signaling [15]. Nevertheless, the role of ABCs within target tissues during autoimmune responses is still poorly understood. In our study, we observed a significant increase in ABCs specifically in the SGs of mature‐adult SjD model mice, but not in age‐matched control mice or young SjD model mice. Moreover, these SG‐infiltrating ABCs may contribute to local inflammation through IFN‐γ production and may also promote their own expansion via autocrine IFN‐γ signaling. These findings suggest that ABCs play a local pathogenic role in autoimmune inflammation during the mature‐adult phase of SjD. In future work, we need to investigate whether ABCs in the SGs contribute to autoantibody production in mature‐adult SjD model mice.

Recent studies have elucidated several mechanisms underlying the differentiation of ABCs. IL‐21 and IFN‐γ have been shown to promote the differentiation of naïve B cells into ABCs [17, 18]. Zinc finger E‐box‐binding homeobox 2 (Zeb2) has been identified as a key transcription factor that not only drives ABC differentiation but also regulates cytokine production by ABCs [19]. Zeb2 expression is upregulated via JAK‐STAT signaling pathways in response to IL‐21 and IFN‐γ stimulation [19, 40]. However, the specific tissue environments in which naïve B cells are exposed to IL‐21 and IFN‐γ and subsequently differentiate into ABCs remain unclear. In addition, it was demonstrated that liver‐specific IL‐21 signaling drives ABC differentiation during LCMV infection [20]. In our study, we found that IL‐21‐producing CD4^+^ T cells were increased in the SGs of mature‐adult SjD model mice. Simultaneously, the gMFI of IFN‐γ was higher in ABCs than in other immune cell populations within the SGs. These findings suggest that IL‐21 from CD4^+^ T cells and IFN‐γ from ABCs may together promote ABC differentiation in the SGs. However, ABCs represented a relatively minor population compared to other IFN‐γ‐producing cells, such as monocytes and Th1 cells. Therefore, further investigation is needed to clarify the extent to which ABCs contribute to the exacerbation of autoimmune pathology in the SGs during SjD.

IL‐21 is predominantly produced by Tfh cells [41]. In a LCMV infection model, interactions between Tfh cells and CD11*c* ^+^ T‐bet^+^ ABCs have been shown to promote antibody production against LCMV [42]. Notably, Tfh cells are also increased in both SjD model mice and patients with SjD [43]. Furthermore, *Il21* gene expression in splenic CD4^+^ T cells and the number of splenic Tfh cells are elevated in SjD model mice compared to control mice [22], suggesting that Tfh cells may facilitate ABC differentiation via IL‐21 in the context of SjD. Interestingly, IL‐21 secretion is also enhanced in Tfh cells from healthy aged human donors compared to younger individuals [44], indicating a potential age‐related mechanism that may contribute to ABC expansion.

The findings in this study suggested that ABC differentiation may be induced by IL‐21 and IFN‐γ within SGs of SjD model mice at the mature‐adult phase. Therefore, it was possible that after naïve B cells migrated into SGs, these cells differentiated into ABCs. Indeed, the serum level of CXCL13, an important factor for the migration of CXCR5^+^ naïve B cells, was upregulated in SjD patients compared to healthy controls [45]. The process by which naive B cells differentiate into ABCs requires further clarification. In addition, T‐bet expression was markedly upregulated in SjD model‐derived B cells compared with control B cells in the in vitro ABC differentiation assay, suggesting that B cells from SjD model mice may intrinsically possess a higher potential for ABC differentiation. Notably, we found no significant differences in the concentrations of IL‐21 or IFN‐γ in the culture supernatants of B cells derived from control and SjD model mice, indicating that B cell‐derived production of these cytokines is unlikely to account for the enhanced ABC differentiation observed in SjD model B cells. These findings raise the possibility that SjD model B cells exhibit increased sensitivity to IL‐21 and IFN‐γ exposure, potentially through elevated expression of cytokine receptors and/or enhanced activation of downstream intracellular signaling pathways. However, the precise mechanisms underlying the heightened susceptibility of SjD model B cells to ABC differentiation remain unclear. In future studies, it will be important to evaluate the expression of IL‐21 and IFN‐γ receptors on B cells, as well as the T‐bet‐associated intracellular signaling pathway.

In conclusion, ABCs were found to infiltrate the SGs of both SjD patients and model mice. The ABC differentiation may be driven by IL‐21 from CD4^+^ T cells and IFN‐γ from several immune cell populations, including ABCs. Our results suggest that ABCs contribute to local inflammation and autoimmune progression in SjD through IFN‐γ‐mediated autocrine signaling, highlighting a potential age‐associated mechanism in disease pathogenesis. Our study indicated that ABC expansion in SGs of SjD model mice began from the mature‐adult phase and continued until the middle‐aged phase. However, it remains unclear whether this finding is similarly observed in SjD patients. Further studies are required to elucidate ABC expansion in SGs of SjD patients.

## Author Contributions

Naozumi Ishimaru and Kunihiro Otsuka supervised the study, acquired the funding, and wrote the original draft. Mari Nishida and Kunihiro Otsuka conceptualized, analyzed data, investigated, and wrote the original draft. Ruka Nagao, Shigefumi Matsuzawa, Aya Ushio, and Takaaki Tsunematsu contributed to the analyzed data and investigated. Ruka Nagao, Shigefumi Matsuzawa, and Aya Ushio equipped mice for analysis. Takaaki Tsunematsu equipped pathological samples of human subjects. Keiko Aota recruited patients.

## Funding

This study was supported by the Japan Society for the Promotion of Science KAKENHI (Grants 23H00438, 23K15976, 22K10172, and 25K22683).

## Disclosure

All authors reviewed the manuscript.

## Ethics Statement

This study was approved by the Committee on Animal Experiments of Tokushima University and Biological Safety Research Center, Japan (Permit Numbers T2022‐70 and T2024‐6) and the Institutional Animal Care and Use Committee of Institute of Science Tokyo (Approval Number A2024‐134A).

## Conflicts of Interest

The authors declare no conflicts of interest.

## Supporting Information

Additional supporting information can be found online in the Supporting Information section.

## Supporting information


**Supporting Information 1** Table S1: Clinical information of non‐SjD sicca and SjD patients. (A) Clinical information of non‐SjD sicca patients. (B) Clinical information of SjD patients.

**Supporting Information 2** Table S2: Clinical information of individual patients. Clinical information (diagnosis, age, gender, focus score, anti‐SSA, and SSB antibodies) are shown. ND: not determined.

**Supporting Information 3** Figure S1: Dynamics of ABCs in SjD model mice. (A) Gating strategy of ABCs using SG cells from control and SjD model mice at the mature‐adult phase. (B) The panel of CD11b and CD95 gated on CD19^+^ CD45^+^ from SGs of SjD model mice at the mature‐adult phase (left). The histogram of T‐bet gated on each CD11b and CD95 expression patterns (right). (C) T‐bet expression in CD11b^+^ CD95^+^, CD11b^+^ CD95^−^, and CD11b^−^ CD95^−^ cells (*n* = 6). (D) Saliva secretion from 6 and 8 weeks of age of control and SjD model mice (*n* = 4). (E) The number of ABCs in SGs from predisease (6 weeks of age, black, *n* = 4), onset (8 weeks of age, orange, *n* = 6) and disease (12–48 weeks of age, red, *n* = 12) stages of SjD model mice. These results shown in this figure are representative data from at least four experiments and are expressed as the mean ± SEMs.  ^∗^ *p* < 0.05,  ^∗∗∗^ *p* < 0.0005,  ^∗∗∗∗^ *p* < 0.00005 (One‐way ANOVA).

**Supporting Information 4** Figure S2: Lymphocyte profile in SGs of control and SjD model mice at middle‐aged phase. (A) FACS panels of CD4 and CD8α (left) and CD4 and CD19 (right) gated on CD45+ 7AAD− cells using SG tissues from control (upper) and SjD model mice (lower) at the middle‐aged phase. (B) The number of CD4^+^ and CD8^+^ T cells (upper) and B cells (lower) in SGs from control (black) and SjD model mice (red) at middle‐aged phase. Data are shown as average ± SEM of mice (*n* = 4).

**Supporting Information 5** Figure S3: IL‐21 and IFN‐γ production by B cells. The concentrations of IL‐21 (A) and IFN‐γ (B) in the supernatants of stimulated B cells from mature‐adult (12 weeks of age) control (black) and SjD model (red) mice were measured by ELISA after 3 days of culture in the presence of anti‐IgM antibody and recombinant mouse CD40L (*n* = 4).

## Data Availability

All the data will be made available by the corresponding author upon reasonable request.
