# Supplementary material for: Tissue‐Specific Expansion of Age‐Associated B Cells via IFN‐γ and IL‐21 Within Salivary Glands in Sjögren Disease
Source: J Immunol Res. 2026 Mar 24;2026:4221251. doi: 10.1155/jimr/4221251 (PMC13140891; doi:10.1155/jimr/4221251)
Supplement: Supplementary file 1 — Supporting Information 1 Table S1: Clinical information of non‐SjD sicca and SjD patients. (A) Clinical information of non‐SjD sicca patients. (B) Clinical information of SjD patients. [file JIMR-2026-4221251-s001.pdf]

Supporting Information 1: Table S1. Nishida M et al.

A

	Age	Female	Anti-SS-A (+)	Anti-SS-B (+)
Non SjD-sicca	55.9 ± 4.3	11/11	4/11	1/11

B

	Focus score	Age	Female	Anti-SS-A (+)	Anti-SS-B (+)
SjD	1	41.2 ± 9.4	5/5	5/5	1/5
	2	56.9 ± 5.0	13/13	13/13	2/12
	3	61.6 ± 6.9	8/8	8/8	1/8
	4	64.0 ± 2.8	10/10	10/10	3/9
	5	61.8 ± 6.4	4/4	4/4	0/4
	6	75.0 ± 0.0	1/1	1/1	1/1
	7	58.0 ± 11.5	3/3	3/3	1/2
