# Supplementary material for: Tissue‐Specific Expansion of Age‐Associated B Cells via IFN‐γ and IL‐21 Within Salivary Glands in Sjögren Disease
Source: J Immunol Res. 2026 Mar 24;2026:4221251. doi: 10.1155/jimr/4221251 (PMC13140891; doi:10.1155/jimr/4221251)
Supplement: Supplementary file 2 — Supporting Information 2 Table S2: Clinical information of individual patients. Clinical information (diagnosis, age, gender, focus score, anti‐SSA, and SSB antibodies) are shown. ND: not determined. [file JIMR-2026-4221251-s004.pdf]

# Supporting Information 2: Table S2. Nishida M et al.

| Patient No. | Diagnosis | Age | Gender | Focus score | Anti-SS-A | Anti-SS-B |
|---|---|---|---|---|---|---|
| 1 | Non SjD-sicca | 33 | Female | 0 | + | – |
| 2 | Non SjD-sicca | 69 | Female | 0 | – | – |
| 3 | Non SjD-sicca | 42 | Female | 0 | + | – |
| 4 | Non SjD-sicca | 50 | Female | 0 | – | – |
| 5 | Non SjD-sicca | 78 | Female | 0 | – | + |
| 6 | Non SjD-sicca | 49 | Female | 0 | – | – |
| 7 | Non SjD-sicca | 67 | Female | 0 | – | – |
| 8 | Non SjD-sicca | 54 | Female | 0 | – | – |
| 9 | Non SjD-sicca | 40 | Female | 0 | + | – |
| 10 | Non SjD-sicca | 69 | Female | 0 | + | – |
| 11 | Non SjD-sicca | 64 | Female | 0 | – | – |
| 12 | SjD | 27 | Female | 1 | + | + |
| 13 | SjD | 38 | Female | 1 | + | – |
| 14 | SjD | 25 | Female | 1 | + | – |
| 15 | SjD | 77 | Female | 1 | + | – |
| 16 | SjD | 39 | Female | 1 | + | – |
| 17 | SjD | 22 | Female | 2 | + | + |
| 18 | SjD | 56 | Female | 2 | + | – |
| 19 | SjD | 31 | Female | 2 | + | – |
| 20 | SjD | 60 | Female | 2 | + | – |
| 21 | SjD | 56 | Female | 2 | + | – |
| 22 | SjD | 80 | Female | 2 | + | – |
| 23 | SjD | 45 | Female | 2 | + | – |
| 24 | SjD | 47 | Female | 2 | + | – |
| 25 | SjD | 81 | Female | 2 | + | – |
| 26 | SjD | 51 | Female | 2 | + | – |
| 27 | SjD | 78 | Female | 2 | + | + |
| 28 | SjD | 67 | Female | 2 | + | ND |
| 29 | SjD | 66 | Female | 2 | + | – |
| 30 | SjD | 30 | Female | 3 | + | – |
| 31 | SjD | 74 | Female | 3 | + | – |
| 32 | SjD | 71 | Female | 3 | + | – |
| 33 | SjD | 74 | Female | 3 | + | – |
| 34 | SjD | 55 | Female | 3 | + | – |
| 35 | SjD | 64 | Female | 3 | + | + |
| 36 | SjD | 70 | Female | 3 | + | – |
| 37 | SjD | 55 | Female | 3 | + | – |
| 38 | SjD | 63 | Female | 4 | + | – |
| 39 | SjD | 81 | Female | 4 | + | + |
| 40 | SjD | 66 | Female | 4 | + | – |
| 41 | SjD | 50 | Female | 4 | + | – |
| 42 | SjD | 69 | Female | 4 | + | + |
| 43 | SjD | 67 | Female | 4 | + | + |
| 44 | SjD | 67 | Female | 4 | + | – |
| 45 | SjD | 54 | Female | 4 | + | ND |
| 46 | SjD | 56 | Female | 4 | + | – |
| 47 | SjD | 67 | Female | 4 | + | – |
| 48 | SjD | 47 | Female | 5 | + | – |
| 49 | SjD | 55 | Female | 5 | + | – |
| 50 | SjD | 72 | Female | 5 | + | – |
| 51 | SjD | 73 | Female | 5 | + | – |
| 52 | SjD | 75 | Female | 6 | + | + |
| 53 | SjD | 46 | Female | 7 | + | + |
| 54 | SjD | 47 | Female | 7 | + | – |
| 55 | SjD | 81 | Female | 7 | + | – |
