# Supplementary figures and images for: Tissue‐Specific Expansion of Age‐Associated B Cells via IFN‐γ and IL‐21 Within Salivary Glands in Sjögren Disease
Source: J Immunol Res. 2026 Mar 24;2026:4221251. doi: 10.1155/jimr/4221251 (PMC13140891; doi:10.1155/jimr/4221251)

Supporting Information 3: Figure S1. Nishida M et al.

A

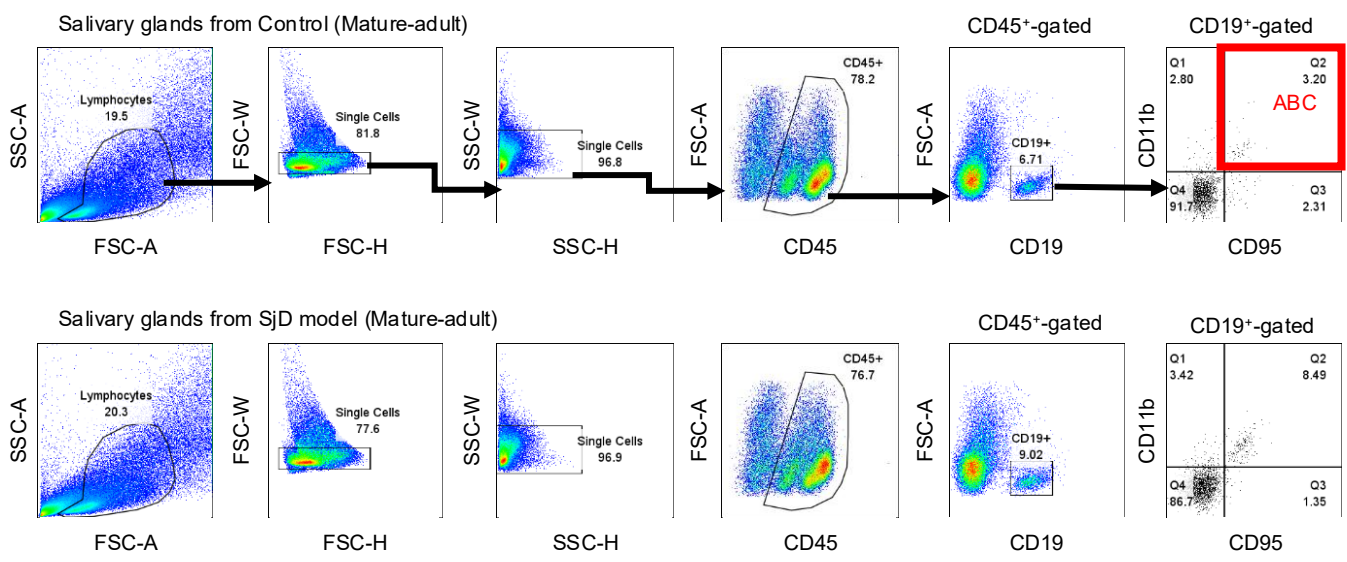

B

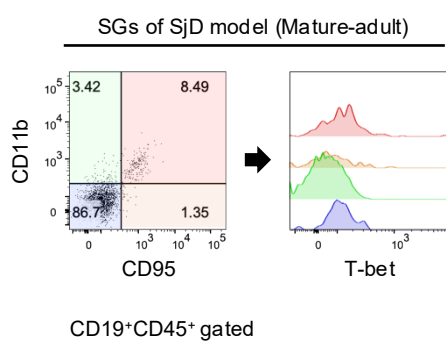

C

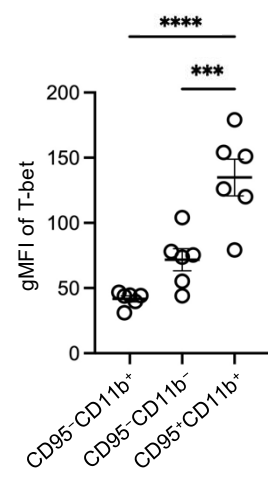

D

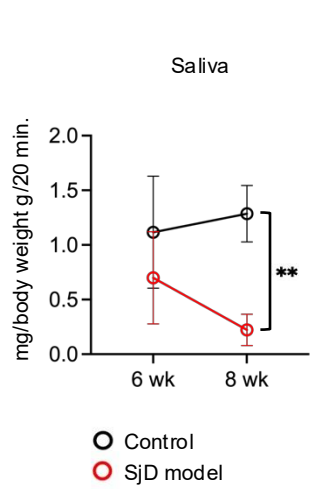

E

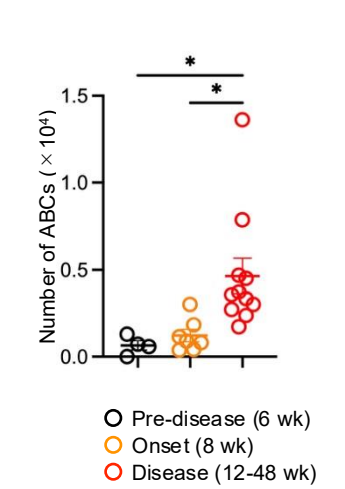

Supplement: Supplementary file 3 — Supporting Information 3 Figure S1: Dynamics of ABCs in SjD model mice. (A) Gating strategy of ABCs using SG cells from control and SjD model mice at the mature‐adult phase. (B) The panel of CD11b and CD95 gated on CD19+ CD45+ from SGs of SjD model mice at the mature‐adult phase (left). The histogram of T‐bet gated on each CD11b and CD95 expression patterns (right). (C) T‐bet expression in CD11b+ CD95+, CD11b+ CD95−, and CD11b− CD95− cells (n = 6). (D) Saliva secretion from 6 and 8 weeks of age of control and SjD model mice (n = 4). (E) The number of ABCs in SGs from predisease (6 weeks of age, black, n = 4), onset (8 weeks of age, orange, n = 6) and disease (12–48 weeks of age, red, n = 12) stages of SjD model mice. These results shown in this figure are representative data from at least four experiments and are expressed as the mean ± SEMs. ∗ p < 0.05, ∗∗∗ p < 0.0005, ∗∗∗∗ p < 0.00005 (One‐way ANOVA). [file JIMR-2026-4221251-s003.pdf]

Supporting Information 4: Figure S2. Nishida M et al.

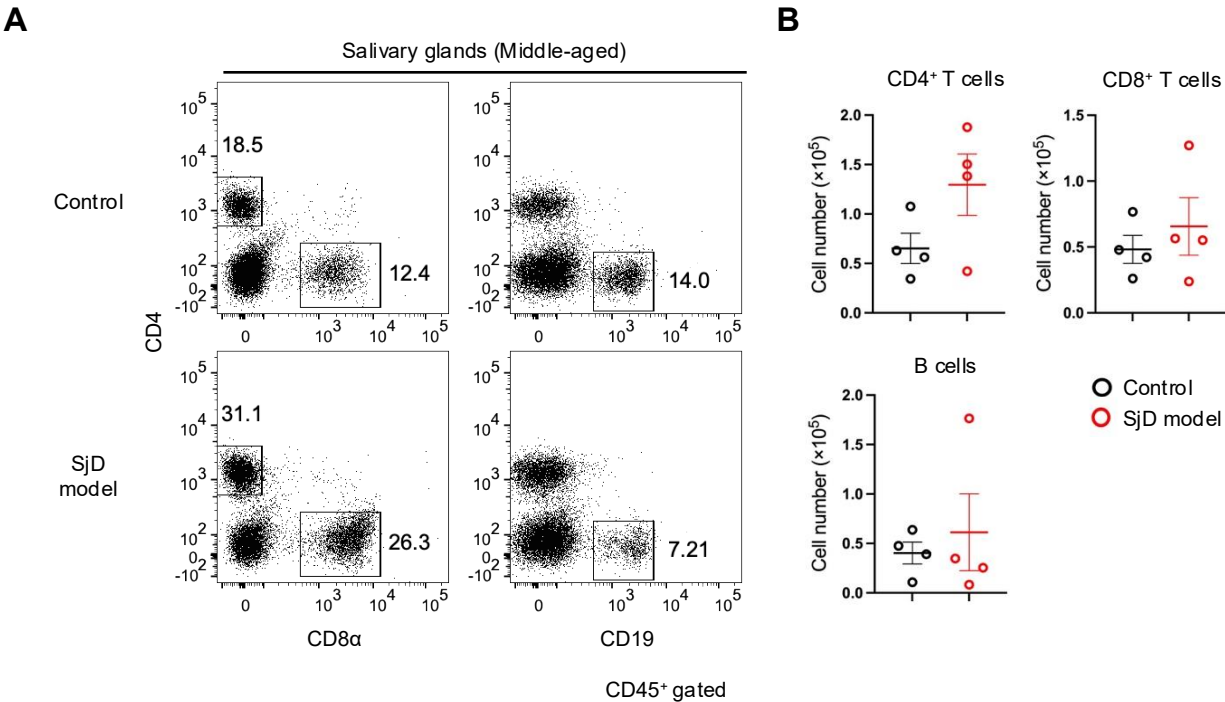

Supplement: Supplementary file 4 — Supporting Information 4 Figure S2: Lymphocyte profile in SGs of control and SjD model mice at middle‐aged phase. (A) FACS panels of CD4 and CD8α (left) and CD4 and CD19 (right) gated on CD45+ 7AAD− cells using SG tissues from control (upper) and SjD model mice (lower) at the middle‐aged phase. (B) The number of CD4+ and CD8+ T cells (upper) and B cells (lower) in SGs from control (black) and SjD model mice (red) at middle‐aged phase. Data are shown as average ± SEM of mice (n = 4). [file JIMR-2026-4221251-s002.pdf]

## Supporting Information 5: Figure S3. Nishida M et al.

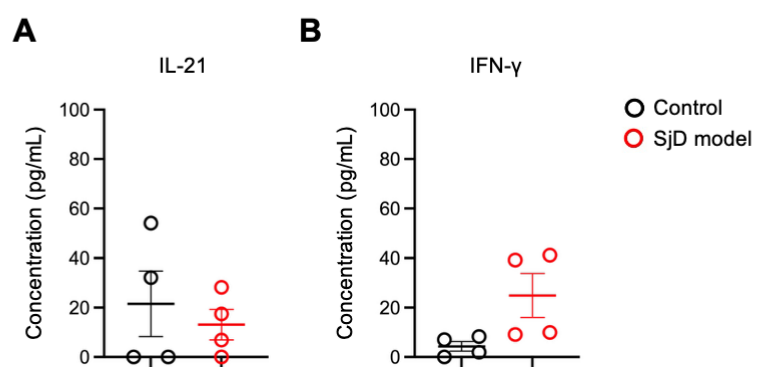

Supplement: Supplementary file 5 — Supporting Information 5 Figure S3: IL‐21 and IFN‐γ production by B cells. The concentrations of IL‐21 (A) and IFN‐γ (B) in the supernatants of stimulated B cells from mature‐adult (12 weeks of age) control (black) and SjD model (red) mice were measured by ELISA after 3 days of culture in the presence of anti‐IgM antibody and recombinant mouse CD40L (n = 4). [file JIMR-2026-4221251-s005.pdf]
